# A Sketch of the Life, Character, and Services of Ambrose Paré

**Published:** 1861-11

**Authors:** S. D. Gross


ART. II.—*A Sketch of the Life, Character, and Services of Ambrose Paré.* By S. D. GROSS, M.D.

To form a just estimate of the services of the man whose character it is my design here briefly to delineate, it is necessary to remember that he entered upon his career at a time when the arts and sciences were nearly extinct, when the intelligence of the world was shut up in the cloister, when philosophy was afraid to speak, and when the people were devoted more to the pursuits of war than to the cultivation of letters and the advancement of civilization. Unable altogether to free himself from the failings and prejudices of his age, he, nevertheless, stands forth as a bright example of true greatness and Christian virtue; performing extraordinary labors as a civil and military surgeon, composing voluminous and useful treatises, occupying the highest social position, and winning for himself, by the force of his intellect, his unwearied industry, and the remarkable purity of his life, an imperishable name. The period in which he lived was one of the most remarkable in history. It was characterized by great and stirring events. At the time of his birth, hardly half a century had elapsed since the invention of the art of printing, and not more than seventeen years since Columbus had announced to Europe the discovery of a new hemisphere, now teeming with thirty millions of people. Ere yet he had fairly entered upon the study of the profession which he afterward so much adorned and illustrated, Luther was sowing the seeds of the Reformation, and shaking the foundation of the religious faith of Europe; Ariosto was maddening his countrymen with the beauty and pathos of his poetry; Erasmus was delighting the world with his matchless scholarship; Copernicus was studying the revolutions of the heavenly bodies; and practical learning and genuine science were beginning, though only beginning, to assert their rights before men.

Ambrose Paré was born at Laval, a little town in the Province of Mayenne, in 1509; consequently a little more than three centuries and a half ago. Of the character and occupation of his parents we are entirely ignorant; all that is satisfactorily known of them is, that they were poor, and that, after having given their son such an education as the opportunities of their residence afforded, they placed him with a priest, who was to instruct him in the Latin language. In consequence, however, of the very small price of tuition, he compelled his pupil to work in his garden, to take care of his mule, and to perform other menial services. It is not improbable that the father of Ambrose originally intended him for some mechanical pursuit, or, it may be, for the ministry, for which his clerical preceptor was perhaps secretly preparing him. After he quitted his service, however, he was placed under the care of a barber-surgeon of Laval, named Vialot, who instructed him in the art of bleeding, and probably also in that of bandaging and of dressing fractures. While thus occupied, accident seems to have determined his future life. Colot, afterward so celebrated as a lithotomist, although then quite young, chancing to have a case of stone in the neighborhood, requested Ambrose to assist in holding the patient, an offer which he eagerly embraced. Struck with admiration at the result of the operation, he resolved to go to Paris to study the higher branches of surgery, an art which was then almost exclusively in the hands of the barbers. Here, availing himself of the services of masters who explained to him the works of Lanfranc, Guido de Cauliac, and John de Vigo, the only surgical text-books of the times, he soon acquired a passion for his profession, and made the most brilliant progress. In a short time after he went to Paris, he was employed by Goupil, a professor in the College of France, to assist him in his practice, attending to such cases as required the minor operations of surgery, as bleeding, bandaging, and the dressing of wounds and ulcers. He also took lessons in the art of elocution, in which, it is said, he made great proficiency, having possessed a natural talent for speaking. It was not long before he obtained a situation in the Hôtel-Dieu, as a resident pupil; and it may well be imagined with what delight and eagerness he availed himself of the opportunities of a hospital which was then, as it has ever since been, one of the largest establishments of the kind in the world. So great was his progress that, after a sojourn here of three years, he was appointed, in 1536, when only twenty-seven years old, to accompany the Colonel-general of artillery, René de Mont-jean, in the capacity of surgeon, to Italy. On his return to Paris, which took place after the surrender of Turin, and the death of his military friend, he entered upon the practice of surgery in that city, and by his skill and good conduct soon attracted the attention of the public.

The opportunities for studying medicine and surgery in the time of Paré can be properly appreciated only by a survey of the state of the profession in the fourteenth and fifteenth centuries. These periods belonged emphatically to the dark ages. The art of printing, invented in 1452, had as yet done little to enlighten the general mind or to advance the interests of our profession. The sun of medicine had long ago sunk behind the horizon; Europe had no medical philosophers; and even the feeble light shed abroad by the school of Salerno, established in the eleventh century, was flickering in its socket and almost extinct. Chemistry, properly so called, had no existence; the only text-book of anatomy was that of Mondino, a medley of inaccuracy and superstition; the practice of medicine was overloaded with polypharmacy; midwifery was exclusively in the hands of females; and in surgery the only works in any favor were those of Lanfranc, Guido de Cauliac, and John de Vigo, all destitute of vigor and originality. The system of instruction by lecturing, as followed at the present day, was entirely unknown. Public teaching was limited to an exposition of the writings of some of the older masters; dissections could be carried on only by stealth; and a student must, indeed, have been exceedingly fortunate if he could occasionally witness an operation upon the living or dead subject.

In the infancy of society medicine and surgery were practiced indiscriminately by the same persons. The human body was considered as a unit, and no distinction was made between internal and external diseases. The separation of medicine and surgery did not occur until a comparatively recent period. Surgery, as such, was originally practiced exclusively by the priesthood, who were thus enabled to exercise a much greater influence upon the public, besides making it a source of profit for the aggrandizement of the church. This state of things continued until 1163, when the Council of Tours, held by Pope Alexander III., resolved to recall the clergy to their legitimate duties, from which they had been partially alienated by their secular occupations, and accordingly decreed that no one who had taken a religious vow should thenceforth engage in the study of medicine under fear of excommunication. Notwithstanding the severity of this penalty, the edict was soon infringed, and it therefore became necessary to fulminate a fresh anathema against transgressors, with the additional proviso that, "as the church abhorred all cruel and sanguinary operations, the priests should not only abstain from the practice of surgery, but also withhold their bendiction from all who professed it in any manner whatever."*

The effect of this new enactment, which took place in the reign of Pope Honorius III., in 1215, was to throw the practice of medicine into the hands of laymen, and to compel the sick to visit the priests at their cloisters. Such of them as were unable to do this contented themselves with sending their dejections, the inspection of which, it was asserted, was quite sufficient for these men to form a correct diagnosis of the disease.

* Millingen's Curiosities of Medical Experience, p. 286. London, 1839.

As to the surgical cases, the priests had them attended by the barbers of the neighborhood, whose chief business it was to bleed, draw teeth, and dress wounds and sores, besides shaving, and tonsurating the heads of their clerical masters. Thus arose the distinction of *barber-surgeon*, a name which they retained until the middle of the seventeenth century.

In 1268, St. Louis, desirous of exhibiting his gratitude to the surgeons for the important services which they had rendered to his soldiers during the Crusades, established at Paris a College of Surgeons, in honor of St. Cosme. To be eligible for membership in this institution, the first of the kind ever founded, it was necessary for the candidate to possess a good academical education, to go through a regular course of studies, and to pass a creditable examination. From the privileges of this college the clerical barbers, or servants of the priests, were, of course, excluded; a circumstance which gave rise to the distinction of the surgeons into two orders, the licentiates of the College of St. Cosme, or the master-chirurgeons, and the shavers, or barber-surgeons, properly so termed. Paré, early in life, became a member of the fraternity of master-surgeons, for in his treatise on wounds, published in 1545, he assumes the title of master-barber-surgeon at Paris, and this title he retained until 1552, when he became surgeon-in-ordinary to King Henry II.

The distinction of master-surgeons and barber-surgeons continued until 1655, when they were incorporated in one college; "a union which was further confirmed, in 1660, by royal ordinance, under some limitations, whereby the barbers should not assume the title of licentiates, bachelors, or professors, nor be allowed to wear the honorable gown and cap that distinguished the higher grades of learning. Red caps were in former times given by each barber to his teacher on his being qualified, and gloves to his fellow-students."

There was another distinction between these two classes of surgeons, and one, indeed, which was common to them and the physicians, namely, the surgeons of the long robe and the surgeons of the short robe, the former constituting, as it were, the dignitaries of the profession. The practitioners of those days were in the habit, in all parts of continental Europe, but more particularly in Paris, of visiting their patients on mules, attired in this peculiar garb, and of concealing their ignorance by holding their conversation in bad Latin; "or, if they condescended to employ the vernacular, mixing it up with such a jargon of scholastic phrase and scientific *technics* as to render it perfectly unintelligible to vulgar ears." This practice continued until after the middle of the seventeenth century, when, chiefly through the influence of Molière, who leveled at it the shafts of his raillery and his sarcasm, it was gradually abandoned for the dress of men of the world. In Great Britain the wig and gold-headed cane, relics of the same pompous custom, were in vogue until a much later period; the latter, indeed, until the death of Dr. Baillie, in 1823, after it had been in use among some of the most illustrious members of the profession in England for a century and a half.

In this country, as well as in certain portions of the continent of Europe, the race of barber-surgeons is not yet wholly extinct. In not a few of the smaller villages and towns of this State some of the barbers, in addition to their distinctive occupation, add that of bleeding, cupping, and extracting teeth, the usual charge for the first and last of these operations being twelve and a half cents; a circumstance which, alone, insures them business.

The pole used by the barbers both in this and other countries was formerly employed by the barber-surgeons as a sign of their particular profession. In blood-letting it was customary, as, in fact, it still is, for the patient to grasp a stick, while the arm was tied up with a red fillet, or a piece of red flannel. When the operation was over, the fillet was twisted round the stick or pole, which was then hung up as a sign, to attract customers, venesection being a very common operation, especially among country people, in the spring and autumn. In time, instead of hanging out the actual pole, a pole painted with red stripes was permanently placed at the door of the barber-surgeon, just as it now is at the shop of the barber.

Thus it will be perceived that the descent of the surgeon was anything but aristocratic, or flattering to his vanity. Like many other pursuits it had its origin in obscurity and humility. Ambrose Paré was its great head, the point of departure of the many illustrious names that have since adorned this branch of the healing art, and conferred so many blessings upon the human race. He was the prototype of a Desault, a Dupuytren, a Hunter, a Richter, a Physick, and a host of other worthies whose fame is as imperishable as the science which they so successfully cultivated.

Paré, as has already been stated, was appointed surgeon-in-ordinary to King Henry II. in 1552, and he served in the same capacity Francis II., Charles IX., and Henry III. These monarchs were all warmly attached to him; he was at once their privy-counselor and their professional adviser; he followed them in their campaigns, and he attended them in their retirement. Their confidence in his skill was boundless. When Charles IX. was thought to be hopelessly ill from the effects of a wound in the arm, consequent upon venesection, Paré soon succeeded in relieving his suffering, and finally in restoring him to health, thus securing his everlasting esteem and gratitude.

The confidence of the public at large in the knowledge and skill of this remarkable man was not less extraordinary. The power of this confidence was strikingly illustrated by a circumstance which happened at the siege of Metz, soon after he was appointed court surgeon. This town, which was invested by Charles V., at the head of a hundred thousand men, contained the flower of the French nobility, and many of the finest soldiers of the day, all reduced to the utmost extremity by hunger and fatigue, hardly any of the wounded escaping death, such was the miserable professional skill of the place. It was at this juncture that the beleaguered army bethought themselves of Paré. They accordingly dispatched a messenger to the French king, entreating him to send Paré to them. He arrived late at night, and being introduced into the city through the treachery of an Italian captain, who received a large reward for his services, both officers and soldiers immediately crowded around him, and, exhibiting the most profound delight at his presence, exclaimed, "we have no longer any fear of dying even if we should be wounded; Paré, our friend, is among us." Every one was inspired with new courage, and the result was that Metz, which, but a moment before, was almost at the point of surrendering, held out until the gallant army, which lay beneath its walls, perished from disease and famine.

His influence over Charles IX. was so great that it enabled him to put a stop to the progress of the massacre of St. Bartholomew, on the 24th of August, 1572, when 70,000 Protestants, better known by the name of Huguenots, were butchered in Paris, and in various parts of France, by the order of that weak and youthful prince. Private animosities, often terminating in bloodshed, had long existed between the Huguenots and Catholics; and affairs had, at length, reached such a crisis that it was determined to exterminate, if possible, the former by a single blow. For this purpose, the court issued orders, on the night above mentioned, to the Provost of Paris, to provide 2000 armed men, each supplied with a particular badge, and to be ready for the work on the ringing of the bell of the palace clock. It is related that, as the awful moment approached, Charles, who was then hardly twenty-three years of age, was seized with horror at the contemplation of the impending scene, and that he would willingly have retracted his orders if he could have withstood the pernicious councils of his mother. His countenance was blanched and ghastly, his forehead was bedewed with cold perspiration, and his whole frame shook like that of a man laboring under an attack of ague. That night the house of every Huguenot in Paris was broken open, and the inhabitants murdered without distinction of age, sex, or rank. The streets were strewed with the bodies of the slain, and the blood-thirsty monarch himself is said to have fired upon his subjects, in their attempt to escape from the Faubourg St. Germain.

For three days and nights this inhuman butchery prevailed in the streets of the French capital; and had it not been for the influence of one man, although that man was a Huguenot, the destruction would probably have been continued until not one Protestant would have been left to tell the sad story of an event which must forever disgrace the name of France. That man was Ambrose Paré. "It was not long," says the Duke of Sully, who has so graphically described this scene of carnage and of cruelty, "before Charles felt the most violent remorse for the barbarous action to which they had forced him to give the sanction of his name and authority. From the evening of the 24th of August he was observed to groan involuntarily at the recitation of a thousand acts of cruelty, which every one boasted of in his presence. Of all those who were about the person of this prince, none possessed so great a share of his confidence as Ambrose Paré, his surgeon. This man, though a Huguenot, lived with him in so great a degree of familiarity that, on the day of the massacre, Charles telling him the time was now come when he must turn a Catholic, he replied, without being alarmed, 'By the light of God, sire, you cannot have forgot your promise, never to command me to do four things, namely, to enter into my mother's womb, to be present in the day of battle, to quit your service, or to go to mass.' The king soon after took him aside, and disclosed to him freely the trouble of his soul: 'Ambrose,' said he, 'I know not what has happened these two or three days past, but I feel my mind and body as much at enmity with each other as if I were seized with a fever; sleeping or waking, the murdered Huguenots seem ever present to my eyes, with ghastly faces, and weltering in blood. I wish the innocent and helpless at least had been spared.' The order which was published the following day, forbidding the continuance of the massacre, was the consequence of this conversation." Paré, at the time of this massacre, was sixty-two years of age, and of all the good deeds which he ever performed there was not one, it may be supposed, that afforded him so much heartfelt satisfaction. Charles did not long survive this horrible war upon his own subjects. His conscience was filled with remorse, and he fell a prey to his infirmities when only twenty-five years of age, being succeeded by Henry III., who had the good sense to retain Paré in his service.

Paré was a copious author. His first publication was given to the world soon after his return from the wars in Italy, under the title of "A Method of Treating Gunshot and other Wounds," the fruits of his observations in the army. It appeared in 1545, when he was not more than thirty-five years of age. This was soon followed by other treatises, both anatomical and surgical, which attracted much attention at home and abroad, and thus served to enlarge a reputation that was already exciting the envy and opposition of his contemporaries, especially the physicians. It was not until 1575 that a complete edition of his works appeared.

The book was printed in folio, and was accompanied by numerous woodcuts illustrative of the anatomy of the human body, of surgical instruments, and of different kinds of monsters, upon the latter of which there is an elaborate and entertaining chapter. The figures, amounting to 300, were prepared at Paré's own expense, at a cost of 3000 livres, a large sum when we consider the character of the professional charges of the times; and they afford an excellent idea of the state of the art of engraving soon after the middle of the sixteenth century.

The edition was very appropriately dedicated to Henry III., "the most Christian king of France and Poland," "a pattern and treasury of my labors." From an epistle accompanying the inscription I make the following extract, as it serves to show how long the author had served his profession and the estimate he placed upon his literary and scientific labors:—

"For God is my witness, and all good men know that I have now labored fifty years with all care and pains in the illustration and amplification of chirurgery, and that I have so certainly touched the mark whereat I aimed, that antiquity may seem to have nothing wherein it may exceed us, besides the glory of invention; nor posterity anything left but a certain small hope to add some things, as it is easy to add to former inventions. In performance whereof I have been so prodigal of myself, my watchings, faculties, and means that I spared neither time, labor, nor cost, whereby I might satisfy and accomplish my own desires, this my great work."

His object evidently was to produce a great and comprehensive treatise, combining both the theory and practice of surgery. In his attempt to carry out this noble design he sought not his own glory alone, but also, as he distinctly avows in the dedication above referred to, the "praise and profit" of the French nation.

In 1582 an edition of his works appeared in Latin, at Paris, beautifully illustrated, and printed in superior style. The name of the translator was not given, but it is generally supposed to have been Hautin, a learned physician, and a personal friend of the author. Not less than six editions of this translation were successively issued at Frankfort, the last in 1652, nearly three quarters of a century after the death of Paré.

From the great celebrity which the works of Paré had attained at home, it is not surprising that they should have been translated into the principal learned languages of Europe. Among the nations which thus appropriated the labors of the illustrious Frenchman, the Dutch deserve the praise of having led the way, a translation having appeared at Leyden as early as 1604. Subsequently four editions were published in Holland, one at Harlem and three at Amsterdam, the last in 1649. Five editions appeared in Germany, and also several in England, the most complete of which is that of Thomas Johnson, at London, in 1634, under the title of "The Works of that famous Chirurgion, Ambrose Paré, translated out of the Latin and compared with the French." The title-page, according to the fashion of that day, is illuminated with a portrait of Paré at the centre of the top, a picture of a man undergoing the operation of trephining being on the left, and an apothecary with his jars and bottles on the right; further down are suspended a skeleton and a subject exhibiting the muscles; while at the bottom of the page are retorts and alembics, surgical instruments, and different animals, the whole forming a very grotesque but interesting feature of the art of engraving in the early part of the seventeenth century. The work passed altogether through three editions, the last bearing the imprint of 1678.

Johnson seems to have executed his translation with great care and fidelity, or, to use his own language, "plainly and honestly, laboring to fit it to the capacity of the meanest artist." The Apology, and Voyages forming the concluding portions of the work, were "Englished" by George Baker, a surgeon, of London, "since that time, as I hear," says Johnson, "dead beyond the seas." The translator has generally confined himself rigidly to his text, but in a few places he has added various explanatory notes.

All these different translations, as well as the original French works of Paré, are now extremely scarce. Occasionally a stray copy, the value of which was not appreciated, or which belonged to some deceased medical man, is found in an auction room, or upon the shelves of some collector of old and rare books; but there are few surgeons of the present day whose libraries are graced by such a treasure.

In 1840, a new edition of his collected works was published at Paris, in three beautiful royal octavo volumes, by Mons. Malgaigne, one of the great admirers of the father of French surgery, and himself a man of great learning and celebrity in his profession. It is illustrated by a full-length portrait of Paré, from a painting by David, and is accompanied by numerous historical and critical notes, preceded by a sketch of the history of surgery in the sixteenth and seventeenth centuries, and a memoir of the life and writings of the author. For this edition, by which I have materially profited in the preparation of this sketch, the profession owes Malgaigne a debt of gratitude, as it places the works of Paré, in a greatly improved form, within the reach of every modern medical scholar.

It was long a question with medical historians, whether Ambrose Paré was really the author of the works which bear his name, or whether he merely superintended their composition. Quite a number of persons have been invested with this honor, as though they had been mere hirelings, selling their labors and their birth-right for a mess of pottage. Malgaigne, who has investigated this subject with his accustomed patience and learning, positively avers that there is no reason whatever to believe that Paré derived any aid of this kind from any one. This indeed appears sufficiently evident from what Paré himself says in the preface to his works; for, after alluding to the fact that he had submitted his writings to the inspection of some of his professional friends, medical as well as surgical, he avows that they are all his own, the materials and the whole fabric being based upon his personal observation and reading.

Much of this dispute respecting the authenticity of Paré's writings doubtless had its origin in the fact that the College of Paris, composed exclusively of physicians, claimed the privilege of licensing books, a privilege which Paré spurned, inasmuch as he had obtained a direct order for issuing his works from the king. The College had evidently not supposed that a surgeon could write so great a treatise upon anatomy, surgery, pharmacy, campaigns, and natural history; its range was far beyond their comprehension, and it was therefore perhaps natural for them, especially as they were extremely jealous of their imagined rights, to accuse Paré of having furnished merely a compilation, the labor chiefly of young physicians, as if young medical men were more competent to compose a treatise on surgery than one who had devoted a whole lifetime to its practice and contemplation.

The greatest service rendered by Paré to his profession and to the world, was his application of the ligature to divided arteries, for the purpose of arresting hemorrhage. The value of this treatment can be duly estimated, at the present day, only by an acquaintance with the practice of the older surgeons in cases of injuries and operations involving any considerable loss of blood. For ages the only remedy for arresting hemorrhage was the actual cautery, or a piece of heated iron applied directly to the orifice of the bleeding vessel. It was particularly popular among the Arabian surgeons, from whom it was borrowed by the practitioners of France, and it was in high repute when Paré entered upon his professional career. Albucasis, who flourished in the latter half of the eleventh century and the beginning of the twelfth, and whose surgical treatises were republished at Oxford, England, as late as 1778, wrote fifty-eight chapters on the actual cautery, in which, among other things, he took special pains to point out its advantages as a hemostatic agent. The result of this practice was that the great majority of those who were subjected to it perished from secondary hemorrhage, coming on at a variable period after the detachment of the sloughs, which almost necessarily followed the searing of the divided vessels. If the artery was large, the unfortunate patient generally died soon after the accident, from the inability of the blood to form a sufficiently strong and opposing clot. In many cases the bleeding recurred from time to time until the system was completely drained of blood. The operation was not only painful, but excessively alarming, so that many persons would almost prefer death to submitting to its cruel requirements. When a limb was amputated, the custom for a long time was to plunge the stump in boiling oil or pitch; and, during naval engagements, it was customary to keep these fluids on hand in the cockpit of vessels, ready for use in case of need. Paré for awhile followed a practice which had been sanctioned by long usage, though he did not fail, from the very first, to perceive its cruelty and utter inadequacy. He was swept along by the general current. His mind, however, was silently engaged in devising a more efficient and scientific treatment; and, after numerous trials, he at length announced to his professional brethren the use of the ligature as a remedy at once safe and of universal applicability. It is possible that he found the traces of his invention in the writings of Celsus and Albucasis, for it is certain that both of these authors refer to the subject, although it does not appear that they ever employed the treatment at the bedside. Indeed, the Roman physician probably never practiced at all, while it is certain that the Arabian continued until the close of his life to confine himself to the hot iron. If this assumption be correct, it follows that the credit of first applying the ligature for the arrest of hemorrhage strictly belongs to Paré, and such, I am satisfied, is the fact. He certainly did not derive the idea from any of his contemporaries; if he had, their clamors would soon have furnished the proof.

It might have been supposed that the truth with which he was to enrich his age would have been first received and fostered in the heart of his professional brethren at Paris. But it was otherwise. Opposition met him in every quarter. Many of his most influential contemporaries became turbulent and furious in their denunciations, and Paré, like Harvey at a later period, for a discovery also pregnant with important blessings, suffered for awhile in his practice. Of his different enemies, none were so loud and vindictive as Gourmelen, President of the College of France, a coxcomb of little brains but great pretension, whom they appointed at a later period to prosecute Paré in the courts of justice, because he dared to publish his work on surgery. "It was then," says this conceited personage, who is now remembered only for his persecutions of his illustrious contemporary, "it was then very forward, rash and presumptuous in a certain individual, to venture upon condemning the cauterization of bleeding vessels after cutting off a mortified limb, a method so highly and continually commended and approved by all the ancients, teaching in opposition to that, without any authority, without knowledge, without experience, without good sense, some new method of his own, of tying arteries and veins."

In the controversies which were thus engendered, coarse and vulgar expressions were often interchanged, imparting a fierceness and a want of dignity not altogether obsolete among certain medical men at the present day. Thus Gourmelen calls Paré a blood-thirsty, cruel rascal, while Paré retorts in language no less vulgar and inelegant. But the illustrious surgeon could not be supposed to be able to submit to every abuse that was heaped upon him by a set of men who, prompted by envy and jealousy, spared no pains to crush him and to destroy his influence.

"You boast, Mons. Gourmelen, that you will teach me my lessons in surgery, and my operations; but in that, I believe, you are a little mistaken, for my education has been quite after another fashion. I have learned my art, not in my closet; no, nor by hearing the discourses of physicians, though that also I have not despised; but in the Hôtel-Dieu, where I lived for three years, seeing many diseases, and practicing many operations upon the living body, and learning also much anatomy by dissection of the dead." "But," continued he, "I have yet more to boast of, for being called into the service of the kings of France, I have, in my time, served four successive kings, having followed them in battles, skirmishes, and assaults; sometimes I have been in sieges, and sometimes shut up with the besieged, curing their wounds." * * * "And last of all, I have lived in this great renowned City of Paris many long years, where, thank God, I have been held in some repute, and ranked, at least, equal with my peers, insomuch that there have been few difficult or celebrated cures in which my head and hand have not been employed. How, seeing these things, dare such a man as you, who has made surgery no part of your study, talk of teaching me?"

These persecutions were excedingly annoying to Paré, whose sensitive mind and proud temper ill qualified him to cope with such adversaries. They declared not only that the application of the ligature was injurious, but that it had been used centuries before, and they almost compelled him to acknowledge his errors by making him seek for traces of his discovery in the writings of Celsus, Galen, Avicenna, and other ancient authors. The result of all this malice and opposition was that few of his contemporaries or immediate successors adopted the practice, which, consequently, soon fell into total neglect. They would rather continue to cauterize arteries and to scald stumps after amputation of the limbs than to have recourse to the ligature. This is so much the more suprising when it is recollected that many of them were eye-witnesses to the success of Paré's practice, and with what care and judgment he laid down the rules for the ligation of the vessels; rules which, with a few trivial exceptions, are still in force in the schools.

Of the value of the ligature in suppressing hemorrhage, it would be folly at the present day to speak, when its claims are so universally established. There is no mode of treatment which can be employed as a substitute; styptics and compression, so much in vogue even as late as the last century, are, as a general rule, wholly inadequate for the purpose; and, as to acupressure, recently introduced to the notice of the profession by Professor Simpson, of Edinburgh, its value as a ready, available hemostatic agent, remains to be determined. Had it not been for this great discovery, it is easy to perceive that many of the operations performed by modern surgeons could never have been attempted without the risk of destroying the patient by hemorrhage. The contemporaries and immediate successors of Paré, by their opposition to the ligature, greatly retarded the progress of surgery, and were thus guilty of the loss of innumerable lives. Paré himself was justly proud of his discovery; but, with the modesty of true genius, and the inspiration of piety, he arrogated nothing to himself, but ascribed all to God. "For the good of mankind, and the improvement and glory of surgery, I was inspired," says he, "with this good thought."

But the application of the ligature for the arrest of hemorrhage was not the only improvement effected by this great man. He was the first to lance the gums in difficult dentition, his own infant daughter being the patient; to employ the twisted suture in the operation for hare-lip; to extract cartilaginous concretions from the knee-joint; and to reduce dislocations of the shoulder by placing the heel in the axilla, a practice so much and so justly in use at the present day.

He was a warm advocate of the use of the bandage, which he applied with much benefit in the treatment of a great variety of affections; and he invented a number of instruments, as well as several ingenious apparatuses. His writings contain the first account of what has since been known as Hey's saw, and of the club-foot boot, claimed to have been first devised by Mr. Syme, of Edinburgh. Among the more curious novelties, as they were doubtless then considered, are delineations of artificial legs, hands, and noses, the latter of which, especially, might perhaps be occasionally beneficially employed at the present day. In short, it is impossible to peruse his works without being struck with the astonishing variety and fertility of his resources.

His skill as an operator must have been very great, for he was an excellent anatomist, and thoroughly trained in the use of instruments. But it was particularly as a therapeutist that his knowledge and sagacity appear to the best advantage. He effected many wonderful cures, both in civil and military practice, and it was this circumstance that gave him such a commanding position among his contemporaries.

As a military surgeon, he was far in advance of his age, both at home and abroad. When he entered the service, he found the army without any regular medical organization, and very few of those that were wounded ever recovered, for all gunshot injuries were at that time, as they had been long previously, regarded as of a poisonous character. Determined to improve the condition of the troops as much as possible, he soon succeeded in deducing order out of confusion, and of placing the service in a position which it had never attained before. The practice then universally was to pour boiling oil into all wounds made with fire-arms. Paré saw that this treatment was not only intensely painful, but that it invariably aggravated the complaint so that very few patients recovered. He accordingly instituted a milder system of medication. During the campaign in Italy, learning that there was a surgeon at Turin celebrated for curing gunshot wounds, he paid him the most assiduous attention for more than two years, in order to obtain from him his famous secret. His career as a military surgeon extended from 1536 down to the battle of Moncontour, in 1569, a period of thirty-three years. No man in the army was ever more beloved, more popular, or more useful.

History furnishes but one similar example of such remarkable popularity. It is that of Larrey, Surgeon-in-Chief of the Grand Army of Napoleon, whom he followed, like a guardian angel, in all his military campaigns, and who pronounced him in his last will the most virtuous man he had ever known. During the retreat from Russia, when the soldiers, exhausted by cold, hunger, and fatigue, were obliged to seek their safety in flight, a river had to be crossed by two temporarily constructed bridges. "But not the troops alone were hurrying over. With the soldiers, and horses, and artillery," says Dr. John Bell, who has so graphically described the scene in his "Medical Heroism," "crowded on unhappy fugitives from Moscow, with their wives, and children, and baggage. In the advancing multitude Larrey was recognized, and immediately a thousand voices exclaimed, 'Let us save him who has saved us. Let him come forward.' All stop at once in their wild rush; Larrey is allowed to reach the bridge, and he is suddenly raised in the arms of the nearest soldiers, and passed from hand to hand, until he is, in this manner, carried entirely across the river. He is saved just at the moment when the bridge gives way, and a crowd of human beings, of both sexes and all ages, together with horses, and cannon, and military wagons, are precipitated into the half-frozen stream beneath, most of them never to reach its banks, never to breathe again."

One of the most interesting portions of Paré's writings is that which relates to his military travels, or, as he terms them, "the voyages made into divers places." This is preceded by an "Apology," in which he attempts, by an appeal to the results of his practice, to defend himself against the aspersions of his enemies respecting the use of the ligature, and nothing could certainly be more satisfactory or triumphant, refuting as he does, completely and thoroughly, all the flimsy and ridiculous charges preferred by their leader, the base and unscrupulous Gourmelen.

The object he had in preparing an account of his travels was to place before the public, in a full and authentic form, his experience as a military surgeon, as well as his observations on the various sieges and battles of which he was an eye-witness He thus foreshadowed a species of literature which has since been so happily elaborated by Larrey in his "Memoirs of Military Surgery, and Campaigns of the French Armies." He details most of the events which he saw with circumstantial minuteness, and, from the remarks which he occasionally drops, it is only too evident that the "Treatise" was written under a kind of necessity, his enemies having openly pronounced many of his oral statements as false, to so shameful an extent did they carry their malice against everything done and uttered by this great man. But all this opposition, here as elsewhere, was in vain. The curs of the profession might bark at and annoy, but could not seriously hurt him. The shafts of their envy recoiled only the more fiercely against themselves.

In performing these military journeys, Paré lost no opportunity of inspecting whatever was of interest in the regions of country through which he passed, and of making the acquaintance of distinguished professional men, with a view of obtaining from them a knowledge of the peculiarities of their practice, and such other information as might be of use to him in the prosecution of his various labors. Like a busy bee, he tried to extract honey from every flower.

Paré never wore the professor's gown and cap, and it was perhaps well he did not; for in those days medical and surgical teaching could certainly not have offered much inducement to any one, much less to a court surgeon.

The personal character of Paré is full of interest. His stature was tall, his figure slender, his countenance grave and dignified. The portrait in his works represents him in his court dress, with a collar on his neck.

Piety formed a prominent trait in his mental constitution, and upon no occasion did he omit to glorify God, always ascribing the wonderful cures which he effected to his immediate aid and interposition. The expression, "I apply the remedy, or I dress the wound, and God cures," occurs in various portions of his writings, and has been inscribed in large letters underneath his statue in the School of Medicine at Paris. In critical cases demanding unusual skill, it was not uncommon for him to pray in private for the success of his remedies. Thus, when he was sent for at a considerable distance from Paris, to attend the Marquis of Auret, who, seven months previously, had been desperately wounded in the lower part of the thigh, and who was then almost exhausted from the effects of numerous consecutive abscesses, before he made any prescription he appealed to Heaven for a blessing upon the means he was about to employ for the relief of his illustrious patient. "Notwithstanding, to give him courage and good hope, I told him," says Paré, "that I would quickly set him on foot, by the grace of God, and the physician's and surgeon's help. Having seen him, I went a walking into a garden, where I prayed to God that he would give me grace to cure him, and that he would give a blessing to our hands and medicaments, to combat against so many complicated maladies."

It is related that he attended without distinction the rich and the poor, and that he made no difference, in this respect, between the Huguenots and Catholics—a circumstance which speaks volumes of praise in favor of his humanity, when we recollect the deadly hatred that existed between these two classes of Christians, he himself espousing the cause of the former.

Although naturally of an amiable disposition, his incessant occupation, both mental and physical, rendered him, at times, irritable; but his anger always speedily subsided, and he never failed, when he thus betrayed his weakness, to ask pardon for the "good old man," the soubriquet by which he was universally known throughout Paris.

In studying the works of Paré, the reader is struck with the air of vanity which pervades their pages, and, at times, is quite obtrusive; the pronoun I is of frequent occurrence, and it is very evident, in many places, that he even likes to magnify his self-importance; but his vanity, after all, is perfectly innocent, for he never praises himself at the expense of others. On the contrary, he always speaks well of his brethren, and sometimes takes special pains to step out of his way to compliment them. He was particularly kind and considerate to young surgeons, especially those who had been his own pupils, doing everything in his power to promote their interest.

Another salient trait in his character was industry. He was never idle; he loved to work, and was never so happy or contented as when he was occupied upon some literary enterprise. From the age of twenty-eight to seventy-three he labored incessantly with his pen. In consequence of the early defects of his education, which his subsequent studies never entirely surmounted, he composed with difficulty, and many of his writings, especially those of his younger days, display an evident want of correct scholarship. A knowledge of the Latin language, then so common among the learned, he never acquired, and he was consequently obliged to avail himself of translations.

His cabinet of curiosities was large, and contained many rare and valuable specimens; among others, a dissected and embalmed human body, a double fœtus strongly resembling the Siamese twins, a uterine mole weighing nine pounds, and the skeleton of an ostrich prepared with his own hands. Like John Hunter, who seems to have inherited many of his great qualities, Paré possessed a great fondness for natural history, and omitted no opportunity to gratify his taste, for we find that his collection comprised contributions not only from Europe, but Asia and Africa. His library was extensive and well selected, and he continued to make additions to it up to the time of his death.

Notwithstanding his expensive outlays, Paré was rich. His lucrative practice, his salary as first surgeon to the king, amounting to 600 livres a year, and the proceeds from the sale of his works, yielded him a large income, enabling him to support a hotel in one of the fashionable streets of Paris, and a handsome country residence in the neighborhood, where, in the midst of his family and his intimate friends, he was wont to spend his nights and his Sabbaths, after the manner of some of our modern surgeons. His disposition was eminently cheerful, and late in life the intervals of his study and toil were often beguiled by watching, with a child-like simplicity and delight, the movements and merrymaking of some little birds, probably canaries, which he had been instrumental in taming. His gayety never forsook him; even amid the horrors of war his pleasantry and mirthfulness served to mitigate the hardships of the soldiers.

Paré had many friends, and no one ever enjoyed in a higher degree the favor of his sovereign, or the confidence of the public. The fact that he was successively surgeon-in-chief to four kings shows how well his moral and professional qualities were appreciated at the French court, in an age when virtue and learning were not always estimated according to their just value. Every one loved the "good old man," and there were few among the great, the wealthy, and the refined who did not consider it a privilege to do him honor. His name throughout Paris, and even in the provinces, was familiar to every one as a household word, and poets delighted to celebrate his virtues in their verses, some of which, with an amiable vanity, he inserted in his published works.

But, although Paré had many friends, he had also numerous enemies; enemies who hated him with a bitter hatred, and who continued their relentless opposition to him to the very borders of the grave. They could never forgive him for the influence which he had obtained over his sovereigns; for the revolution which he effected in the treatment of wounds and operations, by the application of the ligature to the suppression of hemorrhage; for the admirable works which he had produced; and for the elevated social and professional position he enjoyed. They even accused him of having attempted the life of King Henry III. by poison, when, in 1575, that monarch labored under a violent attack of otalgia, accompanied by high delirium, which the ignorant and credulous were not slow to ascribe to the effects of some toxic agent, but which was no doubt due merely to an extension of the disease of the ear to the brain. The belief that the king had been foully dealt with was extensively prevalent, but Paré readily established his innocence by proving that he had applied no remedy except in the presence of the court physicians, several of whom, however, were extremely jealous of his influence, and treated him with the basest ingratitude.

It would be folly to deny that Paré was altogether exempt from the failings of the age in which he lived. He would have been more than man if he could have freed himself completely from the shackles of superstition and credulity which formed such striking features in the human mind of the sixteenth century.

He talks of "monsters occasioned by the craft and subtlety of the devil," and mentions "by what means the devil may deceive us." "Our minds," says he, "involved in the earthy habitation of our bodies, may be deluded by the devils divers ways, for they excel in purity and subtilty of essence, and in the much use of things: they challenge a great pre-eminence, as the princes of this world, over all sublunary bodies. Wherefore it is no marvel if they, the teachers and parents of lies, should cast clouds and mists before our eyes from the beginning, and turn themselves into a thousand shapes of things and bodies, that by these jugglings and tricks they may shadow and darken men's minds." Such sentiments as these need not astonish us when it is remembered that Paré lived in the worst age of the Inquisition, when men almost everywhere believed in magic and witchcraft, when thousands of pilgrims annually visited the Holy Land to expiate their real or imagined sins, and when the Great Reformer, in the quiet recesses of his study, did not hesitate to throw his inkstand into the face of the devil. Our only "marvel" would be if he had been free from them.

His book "of monsters and prodigies," the twenty-fifth of Johnson's edition of his collected works, is a singular medley of erudition, credulity, and superstition, sadly illustrative of the low stock of knowledge in the fifteenth century, and of the gross darkness which enslaved even the best minds of that period. In this book he gives, among many other curious figures, that of an Italian woman who is said to have produced twenty children at two births, nine at the first and eleven at the second; and in the same paragraph he relates, apparently with the most perfect confidence in its truthfulness, the still more remarkable case of the Countess of Virbostans, who brought forth at one accouchement thirty-five living children!

But, notwithstanding these unmistakable evidences of his weakness, it may confidently be asserted that he was far in advance of his time, both in his moral culture, in his professional attainments, and in his practical skill. These defects in his character were the necessary result of the age in which he lived, from whose trammels it was impossible for him wholly to free himself. They formed part and parcel of his mental constitution.

There is but one blot upon the name of Paré, a stain which, as an admirer of this great man, I would fain wipe out, but to which my duty as an impartial historian compels me to advert. Strange to say, Paré himself records the fact, and that too, apparently, without any idea of the blame likely to be attached to it by posterity. The circumstance to which I allude is simply this. At the siege of Hedin, in 1553, Paré, who had been sent with the army, seeing, to use his own language, "about fourscore whores and wenches of the enemies," engaged in drawing water, he prayed the commissary of artillery to discharge his cannon upon that roguish company; but "he made me," says Paré, "much denial, answering me that such kind of people were not worth the powder they should waste. Again I prayed him to level the cannon, telling him the more dead the fewer enemies, which he did, through my request, and at that shot fifteen or sixteen were killed and many hurt." Whether this act was the result of mere wantonness, or of a fanatical idea that he would really be rendering his country an important service, by getting rid of this "roguish company," the mind equally revolts at it, and seeks in vain to reconcile it with the character which Paré had always sustained for piety and benevolence.

As an offset to his conduct on this occasion, may be mentioned the following anecdote, which exhibits his humanity in the most beautiful light. I shall give it in his own words. "A party," says he, "had gone out to attack a church where the peasants of the country had fortified themselves, hoping to get some booty of provisions; but they came back very soundly beaten, and one especially, a captain-lieutenant of the company of the Duke de Rohan, returned with seven gashes on his head, the least of which penetrated through both tables of the skull, besides four sabre wounds in the arm, and one across the shoulder, which divided one-half of the shoulder-blade. When he was brought to the quarters, his master, the Duke, judged him to be so desperately wounded that he absolutely proposed, as they were to march by daylight, to dig a ditch for him, and throw him into it, saying that it was as well that the peasants should finish him. But being moved with pity, I told him," says Paré, "that the captain might get cured. Many gentlemen of the company joined with me in begging that he might be allowed to go along with the baggage, since I was willing to dress and cure him. This was accordingly granted.

I dressed him, and put him into a small, well-covered bed, in a cart drawn by one horse. I was at once physician, surgeon, apothecary, and cook to him, and, thank God, I did cure him to the admiration of all the troops; and out of the first booty the men-at-arms gave me a crown a piece, and the archers half a crown each."

He was a man of the most incorruptible character, and, withal, most warmly attached to his friends, especially his sovereigns, whom he was ever ready to serve in everything that was just and honorable, following them to camp, or remaining at home, as might be most conformable to their wishes. In those days the French court was constantly surrounded by political intriguers, who lost no opportunity to weaken the king's influence with his subjects, or even to destroy his life. It was for the latter purpose that a powerful princess tried to inveigle Paré into her criminal designs, although, knowing the purity of his character, she hesitated a long time before she ventured to reveal to him her wishes. His reply showed the nobility of his soul, at the same time that it administered a most withering rebuke: "Madam," said he, "the idea that you could think me capable of such an act, ought to make me weep the remainder of my days."

Paré spent the evening of his life in tranquillity and resignation, calmly and philosophically awaiting the approach of death. He was still, however, far from being idle, for we find him almost daily engaged in writing corrections on the margins of the fourth edition of his works, and in elaborating his treatise on fevers, which was discovered among his papers long after his death, which occurred on the 20th of December, 1590, in the eighty-first year of his age.

Of his domestic life very little is known. He was twice married, the first union being without offspring, while the second was blessed with two daughters, of whose history, unfortunately, we are ignorant. In 1804, Bonaparte, then First Consul, full of just appreciation of all kinds of merit, instituted special inquiry at Laval with regard to the Paré family, in the hope of being able to render them some service, but without success. It was asserted as late as 1830 that the house once occupied by them at Amsterdam was still to be seen, with the inscription over the door, "here dwelled the descendants of Ambrose Paré," but upon careful search being made no such residence could be found.

The father of French surgery was almost forgotten, or, if not forgotten, hardly ever mentioned, when, in 1812, the Medical Society of Bordeaux proposed, as its prize, a eulogy upon him, which was awarded to Dr. Vimont, and it also projected the publication of a new edition of his works, which, however, was never accomplished. Subsequently the Grand Council of Mayenne proposed the erection of a bronze statue to him at

Laval, after a design by the celebrated artist, Mons. David; but whether this intention was ever carried out or not, my information does not enable me to say.

Paré had two professional contemporaries who, in their respective spheres, occupied nearly the same position as he did in surgery. The first was Fernelius, the restorer of medicine in the sixteenth century, and one of the first physicians who, after Galen, wrote well on the nature and cause of diseases; the other was Vesalius, of Holland, still more celebrated as the father of modern anatomy. In Germany flourished Paracelsus, that compound of science, eccentricity, and charlatanism, which have made his name famous throughout the civilized world. These men were all distinguished by peculiar traits of character which will render their names forever memorable in the history of human exploits.

Fernelius, a Frenchman by birth, after having received a most thorough classical, mathematical, and philosophical education, studied medicine at Paris, and soon distinguished himself as a successful practitioner. His business is reported to have annually yielded him ten or twelve thousand livres, and as he rapidly became rich his enemies did not hesitate to accuse him of avarice. His love of study was excessive. He worked habitually not less than nineteen hours out of the twenty-four; and when one of his friends remonstrated with him on the folly of his conduct, by telling him that such labor and watching would shorten his life, his reply was: "*Longa quiescendi tempora fata dabunt.*" Arnauld, the friend of Pascal, uttered a similar sentiment: "Why don't you rest sometimes?" said his friend, Nicole, to him. "Rest! why should I rest here? haven't I an eternity to rest in?" Fernelius was physician to Henry II. of France, and the author of numerous works, some of which passed through more than thirty editions. Bordeu, his great admirer, thus pithily sums up his character: "Fernelius," says he, "appears like a great light penetrating the thick darkness of his age; never had so elegant an author adorned our chairs; never had genius so gracefully and so ably written on medicine. France has had many great physicians, but none at all comparable to Fernelius."

The history of Vesalius is invested with a melancholy interest. Born at Brussels, in 1514, only five years later than Paré, he early commenced the study of medicine, and rapidly distinguished himself by his passion for dissection, which, on account of the difficulty of procuring subjects, often involved him in many strange adventures. In that age there were no resurrectionists, a body of men so much valued at the present day. Vesalius and his students were obliged to dig up their own subjects, and even sometimes to climb the gibbet in pursuit of the mouldering carcass of the murderer, glad to get any material, however disgusting, to enlarge their anatomical knowledge. Occasionally, as a great favor, criminals were sent from the court and placed at the disposal of the anatomist, with the privilege of dispatching him with poison! Such a statement would not be credited at the present day, if it were not vouched for by one of their own body, Fallopius: "The prince," he says, "ordered a man to be given us, whom we killed in our fashion, and then dissected—*quem nostro modo interfecimus et illum anatomizavimus.*" The article usually employed was opium, in the dose of about two drachms. On one occasion this quantity was administered without any effect, owing, as was supposed, to an attack of intermittent fever. The dose, however, being repeated soon after, the man died. How long this scientific "burking" continued in vogue history does not record.

Vesalius, after having served for two years in the army in the capacity of physician and surgeon, was invited, at the age of twenty-five, to the chair of anatomy in the University of Pavia, where he remained until 1543. He then lectured successively in the schools of Bologna and Pisa, and, in 1544, was appointed physician to Charles V., an office which compelled him to take up his residence at Madrid. Here, in the midst of a most brilliant career, caressed by the court, and beloved by the people, he was seized with nostalgia, which so preyed upon his mind as to induce him to quit Spain. Anxious to conceal from the king the real cause of his distress, he based his resignation upon a desire to visit Jerusalem, in fulfillment of a vow made in early life. Having obtained an honorable discharge, he set out upon his journey, and in due time reached his destination. While there, engaged in contemplating the scenes of the Holy Land, and in studying the character of his fellow-pilgrims, he received the offer of the chair of anatomy in the University of Padua, rendered vacant by the death of his illustrious pupil, Gabriel Fallopius. Glad of an opportunity of returning once more to Italy, he embarked for Venice, but the vessel which carried him was wrecked off the Island of Zanthe, where, in October, 1564, at the age of fifty-eight, he perished from hunger and distress of mind.

Until recently it was generally believed that the real cause of the visit of Vesalius to the Holy Land was an accident which befell him in examining the body of a Spanish nobleman, one of his patients, who died of some obscure disease of the heart. On exposing the chest, this organ, it was asserted, was found to palpitate, thus showing, to the great horror of all present, that life was not entirely extinct. The result was that Vesalius was accused of homicide, and, but for the interposition of the king, who commuted his sentence to a pilgrimage to the Holy Land, he would have been consigned to an ignominious death.

Unfortunately for this story, whose character largely partakes of the romantic, it is entirely destitute of truth. Mons. Burggraeve, Professor of Anatomy in the University of Gand, in his work on the life and writings of Vesalius, published in 1841, and so ably analyzed by Dr. La Roche in the last January number of this journal, has proved the whole charge to be the merest fiction, fabricated by the professional enemies of this great man for the purpose of wounding his feelings and casting reproach upon his reputation. The real motive which induced him to visit the Holy Land remains involved in mystery. Some pretend that the nostalgia of which he complained was a mere pretext to escape from a court for which he had no respect, and in which he was surrounded by jealous and unscrupulous rivals; others assert that his wife, who seems to have been a second Xantippe, was the cause of it. However this may be, no one can help regretting the sad fate which awaited him on his passage home, and which deprived the world of the services of so illustrious an anatomist at a time when they were so much needed.

Vesalius was the first physician, after the commencement of the Reformation, who had the boldness to engage in human dissections, and to make an effort to free anatomical science from the yoke imposed upon it by the ancients. Until his time the authority of Galen and others was regarded as of far greater value than the observation of nature. The man who had the courage to think and act for himself was viewed as a heretic, fit only for the stake. Vesalius, despising the writings of his predecessors, devoted himself incessantly to human dissections, and before he was thirty years of age published his great work on anatomy, illustrated by numerous plates, engraved on wood, from drawings made by the most accomplished artists. A new edition of this immortal production, comprising his treatise on surgery, was issued under the supervision of Boerhaave and Albinus, at Leyden, in 1725, in two splendid folio volumes.

With these illustrious men Paracelsus had few points in common. To talents of a high order, and unquestionable science, he added all the elements and practices of a daring, impudent quack. His very name, in fact, savors strongly of charlatanism. His signature, Philippus Aureolus Theophrastus Paracelsus Bombastus, of Hohenheim, written in full, must have excited the wonder and admiration of many a country man, and served as a flaming advertisement to his boasted skill in the cure of diseases. Born at Einsiedeln, Switzerland, in 1493, he already enjoyed great fame when Paré entered the profession. In 1526, at the suggestion of the learned Erasmus, who had been his patient, and who had conceived the most unbounded respect for his talents and skill, he was appointed Professor of Medicine and Surgery in the University of Basle; and his first official act was to light some sulphur in a brazen chafing dish, and to throw into the flame the works of Galen, Rhazes, and Avicenna, exclaiming, "Sic vos ardebitis in gehennâ!" This act, together with the singularity of his manners, and the boldness of his denunciations of the doctrines of his predecessors, the most enlightened of whom he declared did not know so much as his shoe latchets, rendered him a great favorite with the students. His popularity, however, was of short duration; for a dispute arising between him and the trustees of the school respecting the amount of his salary, he resigned his chair in less than a year, and thenceforth pursued a nomadic, dissolute life, wandering about from village to village, and spending whole nights and days in drinking with the lowest and most debauched persons. In short, he became what, at the present day, would be called a rowdy. Amid all this dissipation, however, he still maintained his reputation by the occasional achievement of extraordinary cures. He boasted that he had invented an elixir by which he could ward off all bodily ailments and indefinitely prolong his life; but while thus vaunting his own praises, he was seized with a fever, of which, in a few days, he died, in the forty-eighth year of his age, thus falsifying his silly and wicked pretensions.

Paracelsus, notwithstanding his dissipations and his many charlatanries, rendered important service to science. He was unquestionably an able chemist and an excellent practitioner, as is proved by the fact that he made a number of important discoveries, among others that of antimony, and that he effected many brilliant cures. His works, which were published after his death in ten quarto volumes, show him to have been a man of great industry and erudition. His treatise on surgery was, in many respects, much in advance of his age. Von Helmont regarded him as a god-send, endowed with vast knowledge and skill, "the forerunner of true medicine," and "the jewel of all Germany;" while Zimmerman declares him to have been a man of the most vulgar tastes and practices, who "lived like a hog, looked like a carter, and found his chief pleasure in the society of the lowest and most debauched of the rabble."

If we cast a retrospective glance at Great Britain, it will be found that she had not one great physician or surgeon during the professional lifetime of Ambrose Paré. Linacre, who died in 1524, after having taken an active part in the institution of the Royal College of Physicians, had given a salutary impulse to medicine; but that impulse was of short duration, for the mantle which he had so long and so gracefully worn did not find one worthy successor. He himself, indeed, left no original additions to the medical literature of his country. His principal contribution was a translation of the works of Galen into Latin, highly esteemed for the purity and elegance of its diction. He was physician to Henry VII. and Henry VIII., founded lectureships in physic at Oxford and Cambridge, and rendered various other important services to medicine, for which his name will always be held in respectful remembrance by Englishmen.

Gradually, however, new light began to break in, and Great Britain effectually aroused from her heavy slumber. The soil which had so long been fallow now produced abundantly, beginning with William Harvey, the discoverer of the circulation of the blood, and Richard Wiseman, a man of real science, a close observer, an able writer, and the greatest military surgeon of the day. These illustrious *savans* opened, as with the magician's wand, the fountains of physiological, medical, and surgical knowledge, and thus imparted an impulse to the healing art, the salutary effects of which cannot be too highly appreciated.

In Italy, during the latter period of the career of Paré, flourished Taliacotius, the great autoplastic surgeon, celebrated for his skill in making new noses, lips, and ears; a man of great science, far in advance of his time. The manner in which he performed his operations was much misrepresented, many alleging that the supplemental structures were borrowed from the arm or breast of another person. The English poet, Butler, sarcastically alludes to the circumstance by observing, in his Hudibras,

> "That when the parent stock died out
> Off dropped the sympathetic snout."

The history of Paré affords many points for reflection. It demonstrates, in the first place, the power of ambition and genius over obscurity of birth, poverty, and defective education; and, secondly, the rewards which follow a life of virtue and of steady devotion to one particular pursuit. His motto, afterward transferred to the title-page of his works, was "labor improbus omnia vincit," and nobly and faithfully did he carry out its behests. No man ever worked harder than he, or with an eye more single to the advancement and honor of the profession which he so ardently loved, and to which, early in life, he resolved to dedicate his whole soul and service. His toils and his fidelity, continued through a period of upwards of sixty years, have earned for him a conspicuous niche in the temple of fame, and the glorious title, accorded to him by the common consent of posterity, of the Father of French Surgery.

> "He was not of an age, but for all time,
> Dear son of memory; great heir of fame."

